# Human Serum Promotes *Candida albicans* Biofilm Growth and Virulence Gene Expression on Silicone Biomaterial

**DOI:** 10.1371/journal.pone.0062902

**Published:** 2013-05-21

**Authors:** Yuthika Hemamala Samaranayake, Becky P. K. Cheung, Joyce Y. Y. Yau, Shadow K. W. Yeung, Lakshman P. Samaranayake

**Affiliations:** Oral Bio-sciences, Faculty of Dentistry, University of Hong Kong, Hong Kong SAR, China; Cornell University, United States of America

## Abstract

**Objectives:**

Systemic candidal infections are a common problem in hospitalized patients due to central venous catheters fabricated using silicone biomaterial (SB). We therefore evaluated the effect of human serum on *C. albicans* biofilm morphology, growth, and the expression of virulence-related genes on SB *in vitro*.

**Methods:**

We cultivated *C. albicans* SC5314 (wild-type strain, WT) and its derivative HLC54 (hyphal mutant, HM) for 48 h in various conditions, including the presence or absence of SB discs, and human serum. The growth of planktonic and biofilm cells of both strains was monitored at three time points by a tetrazolium salt reduction assay and by scanning electron microscopy. We also analyzed by RT-PCR its expression of the virulence-related genes *ALS3*, *HWP1*, *EAP1*, *ECE1*, *SAP1* - *SAP10*, *PLB1*, *PLB2*, *PLC* and *PLD*.

**Results:**

At each time point, planktonic cells of WT strain cultured in yeast nitrogen base displayed a much higher expression of *EAP1* and *HWP1*, and a moderately higher *ALS3* expression, than HM cells. In planktonic cells, expression of the ten *SAP* genes was higher in the WT strain initially, but were highly expressed in the HM strain by 48 h. Biofilm growth of both strains on SB was promoted in the presence of human serum than in its absence. Significant upregulation of *ALS3*, *HWP1*, *EAP1*, *ECE1*, *SAP1*, *SAP4, SAP6 - SAP10*, *PLB1*, *PLB2* and *PLC* was observed for WT biofilms grown on serum-treated SB discs for at least one time point, compared with biofilms on serum-free SB discs.

**Conclusions:**

Human serum stimulates *C. albicans* biofilm growth on SB discs and upregulates the expression of virulence genes, particularly adhesion genes *ALS3* and *HWP1,* and hydrolase-encoding genes *SAP*, *PLB1* and *PLB2.* This response is likely to promote the colonization of this versatile pathogen within the human host.

## Introduction

Central venous catheters (CVCs) fabricated using silicone biomaterial (SB) are widely used for drawing fluids into or from hospitalized patients. These devices have emerged as the most common independent risk factor for implant-associated bloodstream candidal infections, due to their intraluminal or extraluminal colonization by this yeast [1], [2]. *Candida* species, and *Candida albicans* in particular, are the third-leading cause of such CVC-related fungemias [1], [3]. The increased drug resistance of biofilm yeast cells compared to planktonic yeast cells [4] and biofilm-associated transcriptional changes in virulence genes [5] are considered to be the major reasons for such recalcitrant yeast infections.

When a CVC is inserted into a blood vessel, its surfaces are constantly incubated in blood and serum components, including sugars, proteins, electrolytes, and other organic molecules [6], [7]. In this favorable environment, yeast attachment is followed by cell division, hyphal development, and extracellular matrix formation, which leads to development of a loosely packed three-dimensional biofilm structure with fluid channels that permit the exchange of nutrients and waste [8].

Microbial adherence to a substrate, whether biotic (e.g. endothelium) or abiotic (e.g. catheter material), is a prerequisite to biofilm formation [3]. *C. albicans* ability to adhere to substrates is important for virulence, and is mediated through large glycoproteins encoded by genes such as *HWP1* and *ALS* (agglutinin-like sequence gene family) [9]–[12]. The interactions of *C. albicans* with biotic or abiotic surfaces and the subsequent alterations in gene expression have been well studied [13], [14]. Such interactions lead to changes in expression of genes encoding glycosylphosphatidylinositol-dependent cell wall proteins (GPI-CWPs), which mediate adhesion of *C. albicans* to human endothelial cells and epithelial cells; for this reason, GPI-CWPs are also known as adhesins. Furthermore, variations in expression levels of the following virulence-related genes have been described both *in vivo* and *in* *vitro* during biofilm development: adhesion genes *ALS1*, *ALS2*, *ALS3*, *ALS4*, *ALS5*, *HWP1* and *EAP1* [5, 11, 13, 15, and 16] and hydrolase-encoding genes *SAPs* (secreted aspartyl proteases), *LIPs* (lipases) and *PLs* (phospholipases) [17]–[20]. These studies showed that variations in biofilm model system, growth medium and/or other environmental conditions could have a considerable effect on the differential mRNA expression levels of surface-specific genes.

The morphologic transformation among the yeast, hyphal and pseudohyphal forms of *C. albicans* is often considered to be a factor that enhances its virulence. The hyphal phase is thought to promote tissue penetration and colonization of organs during early stages of infection, whereas the yeast form might be important for dissemination in the bloodstream [21]. Previous studies have also demonstrated that genes governing hyphal morphogenesis are co-regulated with those genes encoding virulence factors such as adhesins and hydrolytic enzymes [22]. In addition, yeast morphologic transformations can be induced by serum. However, there is scant information about the role played by human serum or its constituents in *Candida* colonization of CVCs and in their subsequent biofilm growth. Furthermore, any associated changes in expression of virulence-related genes have not yet been evaluated.

To determine the effect of human serum on the development of *C. albicans* biofilms on CVCs (essentially fabricated using silicone biomaterial), we characterized planktonic as well as biofilm growth on silicone biomaterial (SB) with and without human serum, of *C. albicans* SC5314 (a wild-type strain, WT) and its hyphal mutant (HM) (strain HLC54, *efg1/efg1 cph1/cph1*). This mutant strain lacks a functional *EFG1* gene, which encodes a transcriptional regulator that mediates the expression of certain cell wall proteins, such as *HWP1* or *ALS3* [23], [24]. Both of these proteins are important in the yeast-to-hyphae transition and are, therefore, critical for virulence. *EFG1*-deleted strains are known to be growth defective, especially in the yeast phase [25], which may explain our observations above.

In addition, we monitored temporal changes in mRNA levels for genes encoding proteins related to adhesion (*ALS3*, *HWP1*, *EAP1* and *ECE1*) and genes encoding hydrolytic enzymes (*SAP1* - *SAP10*, *PLB1*, *PLB2*, *PLC* and *PLD*) in both strains during planktonic growth *in vitro* and in biofilms developed on SB discs in the presence and absence of human serum.

## Materials and Methods

### *C. albicans* Strains and Growth Conditions

*C. albicans* SC5314 (WT) and its hyphal mutant, (HM) HLC54 (*efg1*/*efg1 cph1*/*cph1*; kindly donated by Prof. NAR Gow, University of Aberdeen, UK) were used throughout the study. The strains were stored in vials with multiple glass beads (Microbank, Pro-Lab Diagnostics, Richmond Hill, Ontario, Canada) at −70°C, subcultured monthly in Sabouraud’s dextrose agar (SDA, Oxoid Ltd., Hampshire, UK) and maintained at 4°C during the experimental period. Yeast nitrogen base (YNB, Difco) medium supplemented with 100 mM glucose was used for liquid cultures. The purity of the phenotypes was confirmed with commercially available API32C identification systems (Biomérieux, Mercy I’Etoile, France) and the germ tube test [26]. YNB medium was supplemented with or without 3% human serum (from male AB plasma, sterile-filtered, H4522, Sigma) throughout this study.

### Preparation of the Standard Yeast Cell Suspension

Prior to each experiment, *Candida* strains were subcultured on SDA at 37°C for 18 h. To prepare the yeast inocula for biofilm growth, a loopful of the SDA culture was transferred into 100 ml of liquid YNB supplemented with 100 mM glucose (Sigma, St. Louis, MO, USA) and incubated at 37°C for 18 h in an orbital shaker (75 rpm). The resulting cells were harvested, washed twice in phosphate-buffered saline (PBS, pH 7.2), centrifuged (4000 × *g*; 5 min) and resuspended in YNB supplemented with 100 mM glucose to a concentration of 10^7^ cells/ml, as assessed by spectrophotometry and confirmed by hemocytometric counting.

### Preparation and Sterilization of SB Discs

Catheter discs of 0.5 cm diameter measured by a micrometer were cut off a catheter (Lily Medical Corporation, Chunan Town, Taiwan) using a lathe (The Colchester Lathe Company Ltd., Colchester, UK). The SB discs were sterilized by immersing in a 0.5% sodium hypochlorite solution for 3 minutes, and they were then washed four times in 100 ml of deionized sterile water for 10 min. The sterility of the discs was verified by rolling the discs on SDA plates, incubating the plates at 37°C for 24 h, and observing the plates for microbial growth.

### Human Serum

Human serum (from male AB plasma, sterile-filtered, H4522) was obtained from Sigma, Aldrich, USA).

### Planktonic Growth

*Candida* strains were grown in SDA medium for 18 h. The resulting yeast cells were suspended in 10 ml of PBS, washed twice by centrifugation at 4,000 × *g* for 5 min, and resuspended in 1 ml of PBS to obtain a dense suspension (equivalent to McFarland standard 4). This yeast suspension was then transferred to 20 ml of YNB supplemented with 100 mM glucose and incubated at 37°C in a water bath at 180 rpm. The cells were harvested following 90 min, 24 h and 48 h incubation by centrifugation at 4,000 × *g* for 5 min. The yeast pellets (approximately 1 ml) were directly used for RNA extraction.

### Biofilm Development of *C. albicans* on Silicone Biomaterial

Biofilms of the two *C. albicans* strains were developed on SB discs as described by Thein *et al*. [27]. The catheter discs were first placed in individual wells of multiwell tissue culture plates (Nunclon Delta, Nunc, Kamstrum, Denmark), into which 1 ml of either 3% human serum solution or sterile distilled water was dispensed, and incubated at 37°C in an orbital shaker (80 rpm) for 1 h. After incubation, the serum solution and the water were aspirated and the serum-coated or control catheter discs were then ready to be immersed into microbial suspensions.

Standard cell suspensions of *C. albicans* strains were prepared at a density of 1×10^7^ cells/ml in YNB supplemented with or without 3% human serum. A 2-ml volume of these cell suspensions was then transferred into selected wells of a pre-sterilized, polystyrene, flat bottom 96-well microtiter plate (IWAKI, Tokyo, Japan), into which the serum-coated or control catheter discs (prepared as described above) were immersed. The plates were then incubated for 90 min at 37°C in an orbital shaker at 80 rpm to promote microbial adherence to the disc. In blank control wells, catheter discs were immersed into 2 ml of YNB.

Samples for biofilm growth analysis were taken at three time points (90 min, 24 h and 48 h) in the following manner. After 90 min (the adhesion phase), the cell suspensions were aspirated, and each disc was washed twice with PBS to remove loosely adherent cells. Then, we added 2 ml of either YNB or YNB supplemented with 3% human serum to each well, and re-incubated the discs at 37°C for 24 h and for 48 h. The plates were then removed from the incubator, and the wells were washed twice with PBS at the respective time intervals to eliminate traces of the growth medium. Finally, we quantified biofilm growth using a tetrazolium salt reduction assay (see below) and investigated biofilm ultrastructure with SEM.

### Quantification of Cell Growth

Biofilm growth was monitored by a metabolic assay based on the reduction of 2, 3-bis(2-methoxy-4-nitro-5-sulfophenyl)-2H-tetrazolium-5-carboxanilide (XTT), a tetrazolium salt [28]. Briefly, XTT (Sigma, MO, USA) was dissolved in PBS to a final concentration of 1 mg ml^−1^. The solution was filter-sterilized using a 0.22-µm pore-size filter and then stored at -70°C until required. Prior to each assay, the XTT solution was thawed and mixed with a freshly prepared 0.4 mM menadione solution (an electron-coupling agent that accelerates the reaction; Sigma, MO, USA) at a volume ratio of 20∶1.

Prior to the XTT reduction assay SB discs with biofilms were washed twice with 2 ml of PBS placed inside the sterile wells of microtitre plates, to remove loosely adherent cells. Afterwards, the biofilm-containing SB discs were transferred into 2-ml plastic vials containing 316 µl of PBS, 80 µl of XTT solution, and 4 µl of menadione solution. After incubation in the dark for 3 h at 37°C, the vials were centrifuged at 13,200 rpm for 10 min; 100 µl of the solution was transferred to a well in a microtiter plate, and the color change in the solution (indicating XTT reduction) was measured at 492 nm using a microtiter plate reader (Spectra-Max 340 Tunable Microplate Reader, Molecular Devices Ltd., Sunnyvale, CA, US). The mean and standard deviation (SD) of the results of three independent experiments were analyzed using a Student’s *t*-test to identify significant differences in biofilm growth at each time point (90 min, 24 h and 48 h).

### Scanning Electron Microscopy

Morphology of planktonic and biofilm cells, as well as the ultrastructure of biofilms, was examined at various stages and under varying conditions of growth by scanning electron microscopy (SEM) as follows. At 90 min, 24 h and 48 h, 100 µls of the planktonic cultures were placed on glass slides and air dried, whereas selected biofilm-containing SB discs were removed from the microtiter plates, fixed in 2% glutaraldehyde for 2 hrs at room temperature. The glass slides and discs were subsequently washed in 70% ethanol, dehydrated in increasing concentrations of ethanol (70% for 30 min, 80% for 30 min, and 95% for 30 min), and stored in 1∶1 hexamethyldisilazane and absolute ethanol in a desiccator. Then, the specimens were air dried and mounted on aluminum stubs with copper tape and coated with gold under low pressure with an ion sputter coater (JEOL JFC1 100: JEOL, Tokyo, Japan). The surface topography of the biofilm was visualized with a scanning electron microscope (Philips XL30CP) in high-vacuum mode at 10 Kv.

## Gene Expression Analysis

### Extraction and Quantification of Total RNA

Planktonic-phase cultures of the *C. albicans* strains were harvested and then washed twice in PBS by centrifugation at 3500 rpm. The yeast pellet was collected for RNA extraction.

Biofilm-containing SB discs were washed three times in PBS to remove loosely adherent cells, and then biofilm cells were recovered from the SB discs by transferring the disc into 1 ml of PBS in eppendorf tubes and vortexing at 180 rpm for 2 minutes to disperse the biofilm. These biofilm cells were collected by centrifugation at 13,200 rpm for 5 minutes.

Total RNA of planktonic and biofilm cells was extracted using an SV Total RNA Isolation system (Promega, Madison, WI, USA) according to the manufacturer’s manual. RNA concentrations were quantified using a Beckman spectrophotometer, and an A_260_/A_280_ ratio between 1.8 and 2.0 ensured RNA purity. Additionally, gel electrophoresis was performed to ensure intact RNA.

### cDNA Preparation and Quantification

Reverse transcription (RT) was performed on 5 µg of RNA in 11 µls of Diethylpryocarbonate water (Sigma, UK). To this mixture, 1 µl of oligo (dT) primer (a concentration of 0.5 µg/µl; Gibco BRL; Life Technologies, Gaithersburg, MD, USA) was added at 70°C for 10 min as previously described by Samaranayake *et al*. [29]. The annealed product was chilled on ice and mixed with 4 µl of ‘first-strand buffer’ (250 mM Tris-HCl, pH 8.3), 2 µl of 0.1 M DTT and 1 µl of 10 mM dNTPs. After incubation at 43°C for 2 min, 1 µl of 200 U µL^−1^ of SuperScript II reverse transcriptase (Gibco BRL; Life Technologies, Gaithersburg, MD, USA) was added to make up a final volume of 20 µl, which was then incubated at 43°C for 90 min to carry out cDNA synthesis [29].

### Quantitative Real-time PCR

Real-time was performed as described in an earlier study [30] to quantify cDNAs (which had been synthesized as described above) corresponding to the following *C. albicans* genes: *EFB1*, *ALS3, EAP1, HWP1, ECE1, SAP1 - SAP10, PLB1, PLB2, PLC* and *PLD*. Forward and reverse primers (listed in Table 1) were designed with Primer Express (Applied Biosystems, Foster City, USA). Real time PCR was carried out with an ABI Step One Real Time PCR System using 2 × SYBR Green Master Mix (Applied Biosystems). The conditions were optimized for 40 cycles of amplification, each cycle consisting of denaturation at 95°C for 15 s followed by annealing at 60°C for 1 min. Quantitation standards, composed of 2-fold serial dilutions of PCR products, were run in conjunction with each set of samples, as well as a master-mix negative control (water). Each experiment was carried out with *EFB1* as the house keeping gene. The tests were performed in duplicate and repeated at least once on different days for reproducibility. A melt curve assay was carried out for each experiment to confirm the specificity of the primers [31]. The relative fold change of each of the virulence genes was ascertained by comparing the gene expression levels of the test and control (with and without human serum) samples.

**Table 1:** Forward and reverse primers used in real-time PCR for the quantification of the expression of *C. albicans* virulence-related genes.

**ALS3-RT-F**	CTG GAC CAC CAG GAA ACA CT
**ALS3-RT-R**	ACC TGG AGG AGC AGT GAA AG
**ECE1-RT-F**	GTC GTC AGA TTG CCA GAA ATT G
**ECE1-RT-R**	CTT GGC ATT TTC GAT GGA TTG T
**EAP1-RT-F**	TGT GAT GGC GGT TCT TGT TC
**EAP1-RT-R**	GGT AGT GAC GGT GAT GAT AGT GAC A
**HWP1-RT-F**	CGGAATCTAGTGCTGTCGTCTCT
**HWP1-RT-R**	CGACACTTGAGTAATTGGCAGATG
**SAPRT1-F**	GAA CCA AGG AGT TAT TGC CAA GA
**SAPRT1-R**	TTT GTC CAG TGG CAG CAT TG
**SAPRT2-F**	GTC ACT TTA AAA AAA CAA GGA GTC ATT G
**SAPRT2-R**	TAT TTG TCC CGT GGC AGC AT
**SAP3RT-F**	CAG CTT CTG AAT TTA CTG CTC CAT T
**SAP3RT-R**	TCC AAA AAG AAG TTG ACA TTG ATC A
**SAPRT4-F**	CGC TGG TGT CCT CTT AGA TTC TG
**SAPRT4-R**	AGG CAT AGA TAA TGC TAC GAG CAA
**SAPRT5-F**	CCA GCA TCT TCC CGC ACT T
**SAPRT5-R**	TTT AGC GTA AGA ACC GTC ACC AT
**SAPRT6-F**	GAT TGT AAA ACT TCA GGT ACC GTT GA
**SAPRT6-R**	CGA AGC AGG AAC GGA GAT CT
**SAP7RT-F**	TTC TCG TGA TGC TGT CCA AG
**SAP7RT-R**	AAA GCC TTC AAA TCC CCA GT
**SAPRT8-F**	GGT GTT AGT AGA GAT CTG GCC ACT ATT
**SAPRT8-R**	GGT GTT CCC ATC AAG ATC ATA AAC T
**SAPRT9-F**	TTC GGG TTC AGG AAC AAC ATC T
**SAPRT9-R**	GCT GAA TGA CGT GTG CTG GTA
**SAPRT10-F**	GGT TTT CGA TAG GCG ATT GAG A
**SAPRT10-R**	CCG TCC TTT TCA GTC TTG AGA TC
**PLB1RT-F**	GGT GGA GAA GAT GGC CAA AA
**PLB1RT-R**	AGC ACT TAC GTT ACG ATG CAA CA
**PLB2RT-F**	TGA ACC TTT GGG CGA CAA CT
**PLB2RT-R**	GCC GCG CTC GTT GTT AA
**PLCRT-F**	AGC CAC CAA TTG GCA AAC TTA
**PLCRT-R**	ACT GCT TGA TTT TAA AGT TGG TTT CC
**PLDRT-F**	TGT TTA CGG TGA AGG GTT GGA
**PLDRT-R**	CAC TGC TAA CCC TTG CTC TCT TG
**EFB1RT-F**	AAG AAG GCT GCT AAA GGT CCA A
**EFB1RT-R**	ATC CCA TGG TTT GAC ATC CAA

### Statistical Analysis

Statistical analysis was conducted by a statistical analysis computer software package (SPSS 20.0 for Windows©, SPSS Inc., Chicago, IL, USA) using the normality test (checked by using the Kolmogorow–Smirnov test) and the equal variance assumptions test (checked by the modified Levene test). T-tests were used to compare a) the biofilm growth of *C. albicans* strains SC5314 (WT) and HLC54 (HM) on silicone biomaterial and, b) expression levels of virulence-related genes in the *C. albicans* strains SC5314 (WT) and HLC54 (HM) on silicone biomaterial in the presence or absence of human serum at three time points (90 min, 24 h and 48 h). A p value less than 0.05 was considered statistically significant.

## Results

### Biofilm Growth of *C. albicans* on SB Discs

Data comparing biofilm growth of *C. albicans* strains SC5314 (WT) and HLC54 (HM) on SB discs in the absence (control) or presence of 3% human serum were obtained by using a tetrazolium salt reduction assay (Table 2). In the WT strain, significant differences in biofilm growth were observed between serum-coated and control SB discs at both 24 h (p<0.001) and 48 h (p<0.01). In terms of biofilm growth significant inter-strain variations between the WT and HM strains were observed in the presence of human serum, with the WT strain demonstrating higher metabolic activity at 24 h when compared to the HM strain at 48 h (p = 0.001) (Table 2).

**Table 2:** Growth of *C. albicans* biofilms on SB discs measured by the XTT reduction assay.

	90 min							24 h							48 h						
	Control^1^		+3% human serum					Control		+3% human serum					Control		+3% human serum				
	Mean^2^	± SD	Mean	± SD	p-value		%	Mean	± SD	Mean	± SD	p-value		%	Mean	± SD	Mean	± SD	p-value		%
SC5314(WT)	0.08	(0.011)	0.26	(0.051)	<0.001	**↑**	236.17	0.50	(0.032)	0.63	(0.063)	0.001	**↑**	26.17	0.68	(0.064)	0.79	(0.056)	0.011	**↑**	15.89
HLC54(HM)	0.07	(0.010)	0.21	(0.050)	<0.001	**↑**	204.33	0.71	(0.106)	0.86	(0.081)	0.024	**↑**	20.26	0.71	(0.069)	0.92	(0.076)	<0.001	**↑**	30.05
**p-value**	0.175		0.309					0.001		0.001					0.366		<0.001				

Strains SC5314 (WT) and HLC54 (HM) were grown in YNB medium with SB discs in the presence or absence of 3% human serum, and growth evaluated for the adhesion phase (90 min) and the biofilm phase (24 h and 48 h). Results are the mean ± SD of three independent experiments.

1 Control : *C. albicans* biofilm developed on silicone biomaterial discs in YNB (without human serum).

2 OD values depicting metabolic activity of *C. albicans* biofilm.

### SEM Studies on Candida Planktonic Growth

**Figure 1:**
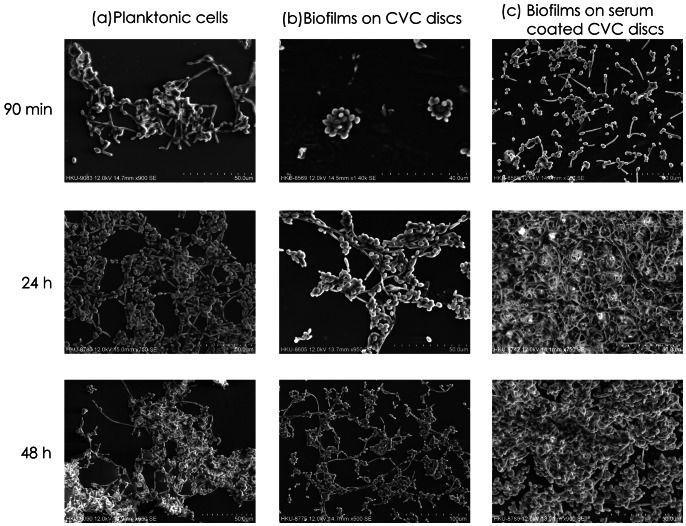
Cell morphology and biofilm ultrastructure of *C. albicans* SC5314 (WT). (a) Planktonic cells incubated in YNB. (b) Biofilms on CVC discs incubated in YNB. (c) Biofilms on serum-coated CVC discs incubated in YNB supplemented with 3% human serum.

**Figure 2:**
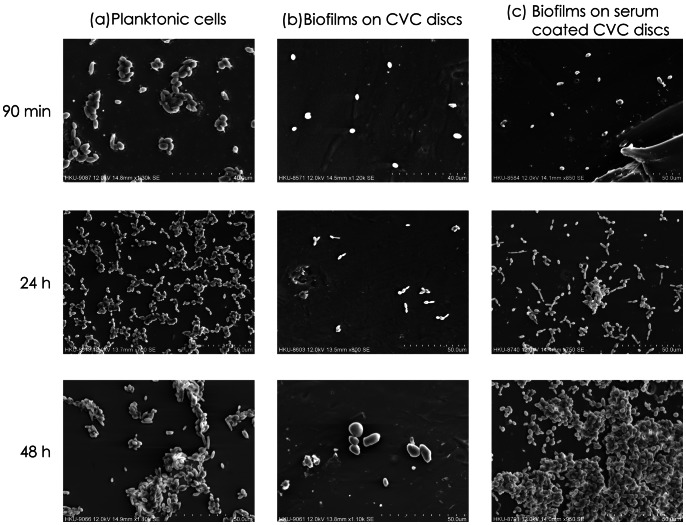
Cell morphology and biofilm ultrastructure of *C. albicans* HLC54 (HM). (a) Planktonic cells incubated in YNB. (b) Biofilms on CVC discs incubated in YNB. (c) Biofilms on serum-coated CVC discs incubated in YNB supplemented with 3% human serum.

After 90 min of incubation, *C. albicans* WT planktonic cells consisted of yeast and germ tubes (Figure 1a), whereas HM planktonic cells were predominantly in the yeast phase (Figure 2a). Following 48 h of incubation, WT cells consisted of co-aggregated yeast cells and a few long strands of hyphae; however, the HM samples at 24 h and at 48 h contained only co-aggregates of yeast cells and they lacked filamentous cells (Figures 1a and 2a).

### SEM Studies on Candida Biofilm Development on SB Discs

During the adhesion phase (90 min), WT *C. albicans* in YNB adhered to the catheter surface and divided to form microcolonies with germ tubes (Figure 1b). At 24 h, the biofilm was characterized by loosely packed yeast and hyphal cells with a thin, heterogeneous architecture. At 48 h, a similar thin biofilm was noted to be devoid of extracellular material (Figure 1b).

WT biofilms on human serum-coated SB discs exhibited numerous germ tubes both at 90 min and at 24 h (Figure 1c). The presence of pseudohyphae and hyphae, covered with a thick extracellular matrix, was evident. A similar biofilm architecture was observed at 48 h, with increased yeast growth (in addition to filamentous forms) (Figure 1c).

By contrast, the HM strain did not seem to form obvious biofilms on SB discs in the absence of human serum (Figure 2b), but it showed sparse biofilm growth in serum-containing YNB at all three time points (Figure 2c). At 24 h, the HM samples exhibited elongated yeast cells and scant growth, whereas large aggregates of cells were observed at 48 h. Furthermore, the biofilm growth of HM at 48 h in human serum, as observed by SEM (Figure 2c), was much lower than that of the WT strain (Figure 1c), thereby confirming the data derived from the tetrazolium salt reduction assay (Table 2).

### Expression of Virulence-related Genes in *C. albicans* Planktonic Cells

The expression levels of various adhesion genes (*ALS3*, *HWP1*, *EAP1* and *ECE1*) and hydrolase genes (*SAP1 - SAP10*, *PLB1*, *PLB2*, *PLC* and *PLD*) for planktonic WT and HM cells was measured at 90 min, 24 h and 48 h using RT-PCR (Table 3).

**Table 3:** Expression levels of virulence-related genes in planktonic cells of *C. albicans* SC5314 (WT) and *C. albicans* HLC54 (HM).

	(A)						(B)						(C)					
	90 min						24 h						48 h					
	HLC54 (HM)		SC5314 (WT)				HLC54 (HM)		SC5314 (WT)				HLC54 (HM)		SC5314 (WT)			
	Mean	Mean	Mean	± SD	p-value		Mean	± SD	Mean	± SD	p-value		Mean	± SD	Mean	± SD	p-value	
*ALS3*	1.08	(0.009)	14.07	(0.852)	***	↑	3.04	(0.163)	3.30	(0.175)	ns	↑	3.91	(0.322)	2.67	(0.144)	**	↓
*ECE1*	0.48	(0.053)	0.64	(0.038)	*	↑	1.69	(0.138)	1.22	(0.052)	**	↓	1.50	(0.068)	0.88	(0.042)	***	↓
*EAP1*	0.60	(0.134)	24.96	(6.355)	**	↑	0.82	(0.072)	39.50	(1.075)	***	↑	2.56	(0.184)	27.99	(1.126)	***	↑
*HWP1*	0.62	(0.108)	421.11	(36.091)	***	↑	0.85	(0.183)	45.80	(2.635)	***	↑	3.00	(0.068)	14.53	(1.048)	***	↑
*SAP1*	0.37	(0.039)	0.70	(0.101)	*	↑	11.63	(1.125)	0.42	(0.021)	***	↓	3.24	(0.274)	2.51	(0.083)	*	↓
*SAP2*	0.37	(0.023)	0.80	(0.021)	***	↑	15.27	(0.846)	0.78	(0.093)	***	↓	3.72	(0.118)	5.95	(0.599)	**	↑
*SAP3*	0.41	(0.094)	0.83	(0.065)	**	↑	19.49	(3.742)	0.37	(0.082)	**	↓	10.46	(2.768)	3.09	(0.220)	*	↓
*SAP4*	0.28	(0.016)	0.69	(0.045)	***	↑	15.66	(1.247)	0.50	(0.013)	***	↓	9.87	(0.694)	5.09	(0.716)	**	↓
*SAP5*	0.47	(0.001)	1.65	(0.048)	***	↑	8.20	(1.749)	1.16	(1.422)	**	↓	3.30	(0.052)	1.48	(0.080)	***	↓
*SAP6*	0.44	(0.020)	1.44	(0.084)	***	↑	11.03	(0.224)	0.45	(0.034)	***	↓	7.54	(0.259)	3.80	(0.250)	***	↓
*SAP7*	0.72	(0.041)	1.84	(0.317)	**	↑	28.01	(8.081)	0.37	(0.114)	**	↓	13.10	(0.113)	4.81	(0.568)	***	↓
*SAP8*	1.40	(0.098)	2.09	(0.052)	***	↑	46.07	(2.286)	0.70	(0.068)	***	↓	11.75	(0.922)	4.77	(0.540)	***	↓
*SAP9*	0.11	(0.027)	0.54	(0.042)	***	↑	2.20	(0.305)	0.23	(0.026)	***	↓	0.97	(0.052)	1.69	(0.061)	***	↑
*SAP10*	0.88	(0.048)	1.76	(0.195)	**	↑	6.32	(0.846)	2.58	(0.248)	**	↓	3.28	(0.137)	4.45	(1.063)	ns	↑
*PLB1*	0.85	(0.161)	11.59	(0.477)	***	↑	57.14	(9.819)	1.63	(0.514)	**	↓	27.60	(6.148)	8.79	(0.640)	**	↓
*PLB2*	0.25	(0.031)	0.61	(0.050)	***	↑	9.69	(2.626)	0.23	(0.034)	**	↓	4.48	(1.009)	2.04	(0.106)	*	↓
*PLC*	0.85	(0.065)	1.41	(0.129)	**	↑	22.17	(0.601)	0.62	(0.083)	***	↓	4.43	(0.118)	2.25	(0.061)	***	↓
*PLD*	0.60	(0.088)	3.02	(0.230)	***	↑	7.52	(1.903)	0.35	(0.018)	**	↓	0.91	(0.068)	0.83	(0.046)	ns	↓

The data were obtained by quantitative real time RT-PCR at three time points (90 min, 24 h and 48 h). Results are the mean ± SD values of three independent experiments conducted on different days.

ns : p>0.05;

* p<0.05;

** p<0.01;

*** p<0.001.

The arrows refer to up/down regulation of mRNA expression levels of virulence-related genes between the wild type *C. albicans* SC5314 (WT) and its corresponding hyphal mutant HLC54 (HM).

#### a) Adhesion genes

The mRNA levels of the adhesion genes *EAP1* and *HWP1* were significantly higher in WT planktonic cells than in HM planktonic cells at all three time points (p<0.01) (Table 3). Transcript levels for *ALS3* and *ECE1*, however, were in all cases low and did not show significant differences between the two strains (Table 3).

#### b) SAP genes

After 90 min of incubation, the expression of all ten *SAP* genes studied was significantly higher in WT planktonic cells than in HM planktonic cells (Tabel 3). Interestingly, however, at 24 h, the expression levels of all ten *SAP* genes became significantly lower in the WT strain than in the HM strain (p<0.001). After 48 h of incubation, most of the *SAP* genes (*SAP1* and *SAP3 - SAP8*) exhibited lower transcript levels in WT cells than in HM cells, whereas the opposite was true for *SAP2, SAP9* and *SAP10* (Table 3).

#### c) PL genes

Significantly higher mRNA levels for all four phospholipase genes (*PLB1, PLB2, PLC* and *PLD)* were detected at the 90-min time point in WT cells in comparison to HM cells (p<0.001) (Table 3). However, all these genes were significantly downregulated in the WT strain at 24 h and 48 h of incubation (p<0.05), whereas they were upregulated (especially *PLB1*) at the same time points in the HM strain (Table 3).

### Expression of Virulence-related Genes in *C. albicans* Biofilms on SB

#### a) Adhesion genes

For biofilms of the WT strain, levels of the *ALS3* mRNA were moderately higher at 24 h, but was significantly higher at 48 h (p<0.001), in human serum-coated SB discs than in the serum-free controls (Table 4). A stimulatory effect of human serum on *ALS3* expression was also observed for the HM strain at 90 min and at 48 h (Table 4). *ECE1* mRNA levels of the WT biofilms were significantly higher at all three time points (p<0.05) in human serum-incubated SB discs than in the serum-free controls. A significant increment in *ECE1* mRNA levels was also observed, but only at 24 h, for HM biofilms on human serum-incubated SB discs compared with the control (p<0.05) (Table 4).

**Table 4:** Expression levels of virulence-related genes in biofilms of *C. albicans* SC5314 (WT) grown on SB discs in the presence or absence of human serum.

	(A)						(B)						(C)					
	90 min						24 h						48 h					
	YNB		YNB+3% human serum				YNB		YNB+3% human serum				YNB		YNB+3% human serum			
	Mean	± SD	Mean	± SD	p-value		Mean	± SD	Mean	± SD	p-value		Mean	± SD	Mean	± SD	p-value	
*ALS3*	2.45	(0.190)	1.92	(0.297)	ns	↓	1.63	(0.119)	2.01	(0.282)	ns	↑	0.70	(0.035)	0.90	(0.012)	**	↑
*ECE1*	11.84	(1.025)	20.08	(2.551)	**	↑	2.23	(0.483)	5.26	(0.327)	***	↑	2.56	(0.312)	3.18	(0.160)	*	↑
*EAP1*	0.36	(0.017)	0.31	(0.015)	*	↓	0.72	(0.088)	0.90	(0.034)	*	↑	0.73	(0.051)	0.95	(0.168)	ns	↑
*HWP1*	16.83	(1.151)	13.11	(0.031)	**	↓	1.10	(0.233)	1.04	(0.130)	ns	↓	0.52	(0.042)	0.92	(0.178)	*	↑
*SAP1*	0.10	(0.006)	0.15	(0.005)	***	↑	0.49	(0.012)	0.76	(0.003)	***	↑	0.86	(0.053)	1.62	(0.433)	*	↑
*SAP2*	0.31	(0.004)	0.23	(0.021)	**	↓	1.01	(0.028)	1.59	(0.129)	**	↑	1.43	(0.165)	1.82	(0.175)	*	↑
*SAP3*	0.17	(0.037)	0.22	(0.023)	ns	↑	0.79	(0.185)	1.00	(0.103)	ns	↑	0.76	(0.073)	1.02	(0.184)	ns	↑
*SAP4*	0.09	(0.012)	0.21	(0.039)	**	↑	0.54	(0.023)	1.03	(0.033)	***	↑	0.97	(0.124)	1.56	(0.019)	**	↑
*SAP5*	0.11	(0.021)	0.14	(0.007)	ns	↑	0.59	(0.047)	0.72	(0.035)	*	↑	0.75	(0.087)	0.88	(0.085)	ns	↑
*SAP6*	0.12	(0.004)	0.18	(0.018)	**	↑	0.58	(0.081)	0.81	(0.082)	*	↑	0.80	(0.061)	1.21	(0.021)	***	↑
*SAP7*	0.56	(0.039)	0.40	(0.064)	*	↓	0.52	(0.182)	0.61	(0.041)	ns	↑	0.54	(0.123)	0.81	(0.133)	ns	↑
*SAP8*	0.21	(0.016)	0.39	(0.064)	*	↑	0.74	(0.035)	1.14	(0.071)	**	↑	1.01	(0.199)	1.78	(0.230)	*	↑
*SAP9*	0.20	(0.019)	0.25	(0.018)	*	↑	0.62	(0.013)	0.67	(0.017)	*	↑	1.31	(0.088)	1.62	(0.134)	*	↑
*SAP10*	0.45	(0.016)	0.56	(0.069)	*	↑	0.09	(0.011)	0.71	(0.091)	***	↑	1.39	(0.125)	2.01	(0.159)	**	↑
*PLB1*	0.18	(0.061)	0.17	(0.012)	ns	↓	0.69	(0.064)	0.90	(0.077)	*	↑	0.68	(0.090)	0.77	(0.148)	ns	↑
*PLB2*	0.06	(0.008)	0.08	(0.002)	*	↑	0.27	(0.051)	0.35	(0.035)	ns	↑	0.40	(0.035)	0.47	(0.081)	ns	↑
*PLC*	0.19	(0.006)	0.25	(0.010)	**	↑	0.59	(0.044)	0.54	(0.032)	ns	↓	0.72	(0.020)	0.62	(0.098)	ns	↓
*PLD*	0.38	(0.036)	0.44	(0.055)	ns	↑	0.82	(0.237)	1.18	(0.223)	ns	↑	0.81	(0.043)	1.39	(0.178)	**	↑

Samples were taken during the adhesion phase (90 min) and biofilm growth (24 h and 48 h). Results are the mean ± SD values of three independent experiments.

ns : p>0.05;

* p<0.05;

** p<0.01;

*** p<0.001.

The arrows refer to up/down regulation of mRNA expression levels of virulence genes of *C. albicans* SC5314 (WT) in the presence of human serum relative to the mRNA expression levels devoid of serum.

In contrast to *ALS3* and *ECE1* transcript levels, human serum did not seem to have a significant effect on mRNA levels of *EAP1* and *HWP1* in SB biofilms of either strain (Table 4). In WT biofilms, the *HWP1* mRNA levels were very high at 90 min and were drastically downregulated at 24 h and 48 h on SB discs with or without serum, while in HM biofilms *HWP1* mRNA levels were negligible under all test conditions (Table 4).

In summary, transcript levels of the adhesion genes in the HM biofilms were different from those in the WT biofilms. In the HM strain, there was a significant upregulation of *ALS3*, *ECE1* and *EAP1* transcripts in serum-coated SB discs at 90 min (p<0.01). However, at 24 h, only *ECE1* expression was upregulated, and the other transcripts were downregulated (p<0.001). To conclude, the mRNA expression levels of the four adhesion genes in WT strain biofilms in serum coated discs were comparatively higher than the HM strain biofilms (Table 4).

#### b) SAP genes

Transcript levels for six of 10 *SAP* genes (*SAP1, SAP4, SAP6, SAP8*–*SAP10*) were significantly higher at all three time points in the WT biofilms from human serum-treated SB discs than in the serum-less WT controls (p<0.05) (Table 4). However, WT biofilms on control discs demonstrated significantly higher levels of transcripts for *SAP2* and *SAP7* at 90 min. (p<0.05), lower levels of *SAP3* and *SAP7* transcripts at 24 h, and lower levels of *SAP3, SAP5* and *SAP7* transcripts at 48 h, compared with biofilms on human serum coated discs (Table 4).

The levels of six *SAP* genes (*SAP1, SAP2, SAP4, SAP5, SAP6* and *SAP8*) were higher in HM biofilms on human serum-coated SB discs than in HM controls at 90 min. The expression of seven *SAP* genes (*SAP2–SAP6, SAP8* and *SAP10*) was also significantly higher in HM serum-coated biofilms than in the HM controls both at 24 h and at 48 h (p<0.005) (Table 5).

**Table 5:** Expression levels of virulence-related genes in biofilms of *C. albicans* HLC54 (HM) grown on SB discs in the presence or absence of human serum.

	(A)						(B)						(C)					
	90 min						24 h						48 h					
	YNB		YNB+3% human serum				YNB		YNB+3% human serum				YNB		YNB+3% human serum			
	Mean	± SD	Mean	± SD	p-value		Mean	± SD	Mean	± SD	p-value		Mean	± SD	Mean	± SD	p-value	
ALS3	0.29	(0.026)	0.38	(0.026)	*	↑	1.17	(0.152)	1.15	(0.056)	ns	↓	0.62	(0.021)	0.80	(0.019)	***	↑
*ECE1*	0.60	(0.134)	0.84	(0.009)	*	↑	0.82	(0.072)	2.09	(0.216)	**	↑	2.56	(0.184)	1.59	(0.184)	**	↓
*EAP1*	0.17	(0.005)	0.21	(0.013)	**	↑	0.66	(0.062)	0.75	(0.025)	ns	↑	0.99	(0.054)	0.98	(0.047)	ns	↓
*HWP1*	0.28	(0.053)	0.37	(0.082)	ns	↑	0.14	(0.016)	0.40	(0.047)	**	↑	0.23	(0.007)	0.15	(0.062)	ns	↓
*SAP1*	0.26	(0.008)	0.42	(0.038)	**	↑	1.02	(0.078)	1.29	(0.227)	ns	↑	1.40	(0.145)	1.42	(0.042)	ns	↑
*SAP2*	0.51	(0.018)	0.61	(0.052)	*	↑	0.72	(0.046)	0.91	(0.018)	**	↑	1.00	(0.079)	1.32	(0.144)	*	↑
*SAP3*	0.71	(0.073)	0.78	(0.054)	ns	↑	0.84	(0.260)	1.29	(0.033)	*	↑	1.56	(0.129)	1.67	(0.233)	ns	↑
*SAP4*	0.33	(0.013)	0.55	(0.023)	***	↑	0.86	(0.019)	1.67	(0.099)	***	↑	1.11	(0.006)	1.44	(0.029)	***	↑
*SAP5*	0.43	(0.008)	0.71	(0.032)	***	↑	0.37	(0.034)	0.72	(0.017)	***	↑	0.46	(0.058)	0.65	(0.045)	*	↑
*SAP6*	0.42	(0.023)	0.73	(0.013)	***	↑	0.63	(0.056)	1.12	(0.070)	**	↑	0.79	(0.022)	0.98	(0.081)	*	↑
*SAP7*	1.86	(0.051)	1.75	(0.258)	ns	↓	0.82	(0.052)	2.49	(0.335)	**	↑	0.85	(0.133)	0.91	(0.031)	ns	↑
*SAP8*	0.79	(0.051)	1.21	(0.063)	**	↑	2.41	(0.037)	2.91	(0.279)	*	↑	1.60	(0.242)	2.33	(0.259)	*	↑
*SAP9*	0.50	(0.025)	0.82	(0.296)	ns	↑	0.96	(0.072)	0.90	(0.044)	ns	↓	1.54	(0.063)	2.33	(0.383)	*	↑
*SAP10*	2.10	(0.376)	2.30	(0.222)	ns	↑	0.87	(0.029)	1.14	(0.104)	*	↑	1.38	(0.104)	2.66	(0.218)	**	↑
*PLB1*	0.24	(0.015)	0.46	(0.121)	*	↑	1.54	(0.182)	3.43	(0.137)	***	↑	1.93	(0.147)	1.49	(0.079)	*	↓
*PLB2*	0.31	(0.054)	0.38	(0.093)	ns	↑	0.56	(0.116)	0.74	(0.053)	ns	↑	0.61	(0.121)	0.64	(0.017)	ns	↑
*PLC*	1.00	(0.074)	1.05	(0.088)	ns	↑	2.28	(0.529)	1.79	(0.216)	ns	↓	0.71	(0.054)	1.04	(0.079)	**	↑
*PLD*	1.12	(0.082)	1.35	(0.107)	*	↑	1.39	(0.331)	1.31	(0.123)	ns	↓	0.93	(0.133)	2.44	(0.476)	**	↑

Samples were taken during the adhesion phase (90 min) and biofilm growth (24 h and 48 h). Results are the mean ± SD values of three independent experiments.

ns : p>0.05;

* p<0.05;

** p<0.01;

*** p<0.001.

The arrows refer to up/down regulation of mRNA expression levels of virulence genes of *C. albicans* HLC54 (HM) in the presence of human serum relative to the mRNA expression levels devoid of serum.

#### c) PL genes

*PLB1* transcript levels in serum-treated WT biofilms were higher than the levels in serum-free controls both at 24 h and 48 h time points. By contrast, *PLB1* expression in serum-treated HM biofilms was significantly much higher than in the controls both at 90 min and 48 h (p<0.001) (Table 5).

Both up- and downregulation of *PLB2, PLC* and *PLD* mRNA transcripts during biofilm growth for both the WT and HM strains in human serum-coated/uncoated SB discs during the total incubation period were evident (Table 5). These results demonstrate that the effect of serum on gene expression was variable, depending on time point and strain.

## Discussion

### *Candida albicans* Biofilms on Central Venous Catheters

Following insertion into blood vessels, CVC surfaces become covered with a film of proteins, sugars, electrolytes and other blood components that promote the development of a biofilm; a complex microbial community of bacteria and yeasts enveloped in an extracellular matrix of proteins and polymeric material [32]. Indeed, electron microscopy studies have demonstrated the abundance of biofilm-associated microorganisms on most, if not all, CVCs [33].

Biofilm features can be reproduced to some extent in *in vitro* experimental models pre-conditioned with host proteins [34]. Previous reports have demonstrated that *in vitro* growth of *Candida* biofilms can be affected by serum components [35], [36], and that human serum promotes and modifies biofilm growth of *C. albicans* in particular by initiating germ-tube production [37]. Increased biofilm growth of *C. albicans* has also been reported on catheter surfaces pre-conditioned with body fluids, such as serum and blood [1], [36]. Many have reported that such biofilm activity could be reliably quantified using the XTT assay provided appropriate controls are used [28]. In the current study, we set up to assess the effect of human serum on the formation of *C. albicans* biofilms on SB discs *in vitro*. We also aimed to determine how human serum affects the differential expression of *C. albicans* virulence-related genes (encoding adhesins, hyphae and extracellular hydrolases) in such biofilms. For this purpose, we used a *C. albicans* WT strain and a derived HM strain. In contrast to the wild type *C. albicans* SC5314, the double mutant *efg1/efg cph1/cph* neither produces hyphae nor invades a reconstituted human oral epithelium model supporting the theory that the hyphal phase in an important virulence attribute of *C. albicans* isolates [38]. Hence, we examined the HM strain in addition to the WT strain to investigate if human serum modifies the virulence of *Candida* by virtue of its hyphal appendages.

### *C. albicans* Biofilm Growth on Silicone Biomaterial

In our *in vitro* study, we noted a thick biofilm on human serum-coated SB discs only in the WT and not the HM strain during a 48 h incubation period. These biofilms consisting of a dense network of filamentous cells embedded in an extracellular matrix have been previously documented on surfaces such as denture acrylic or polystyrene [39], [40]. Furthermore, we found that human serum stimulated cell proliferation and filamentation in WT biofilms grown on SB discs. Indeed, the serum-free SB discs showed scant growth of WT cells during the initial 90 min, although, filamentation was also observed after 24 h. Our findings are consistent with those of an earlier study that showed a role for serum in the increased formation of hyphae in *C. albicans* biofilms developed on microtiter plates and catheters [13].

We observed minimal adhesion of the HM strain to SB discs and it was completely defective in filamentous growth in the absence of human serum throughout the 48 h incubation period. The HM strain displayed pseudo-hyphal growth on serum-coated SB discs, both at 24 h and 48 h, thus indicating the powerful effect that human serum exerts on yeast filamentation.

*C. albicans* biofilms, when grown on static denture acrylic surfaces, have a thickness of 25 µm [39], whereas those grown using an *in vitro* model with a flowing growth medium can have a thickness of up to 70 µm [41]. Interestingly, the thickness of biofilms on a central venous catheter has been shown to exceed 100 µm *in vivo* in a rat model [42], the authors attributed this increased thickness to the flow characteristics of the model and to the host-derived conditioning film covering the device. Fully mature biofilms developed within 24 h in this rat model and were composed of a dense multi-layered network of yeast cells and hyphae [42].

### *C. albicans* Virulence Gene Expression on Silicone Biomaterial

Virulence-related gene expression in biofilms has predominantly been investigated in bacterial infections [43], and little is known about virulence gene expression in fungal biofilms. However, there are several studies available on the differential expression of various mRNAs and some studies on the transcriptomic analyses during *Candida* adherence and hyphal formation *in vitro* and *in vivo* [7], [13], [42], [44]–[46].

### Adhesion Genes

In our experiments, the expression of *ALS3,* a member of the *ALS* gene family, was low during planktonic growth in the WT strain. Nevertheless, the *ALS3* gene was highly expressed in the WT strain at all three time points in the SB discs incubated in human serum. It appears, therefore, that *ALS3* is important both for adhesion and for biofilm growth of *C. albicans* on SB discs and that human serum enhances the upregulation of *ALS3* expression. Furthermore, a significant increase in *ALS3* expression in WT biofilms at 48-h, when compared with the HM strain, suggests that *ALS3* is important for biofilm maturation. Previous studies have demonstrated the importance of *ALS3* expression in biofilm formation on silicone substrates [45, 47 and 48]. Similar expression patterns of *ALS3* have been revealed by RT-PCR of RNA samples from *C. albicans* biofilms developed on reconstituted human buccal epithelium, denture acrylic, silicone-elastomer catheter material and other abiotic surfaces [49], thus suggesting that this gene has a role in the formation of biofilms on diverse surface types. Furthermore, expression of *ALS3* (and *ALS2*) and *HWP1* has been observed in biofilms associated with abiotic surfaces [5, 13, 45 and 50]. In general, our findings are consistent with the previous studies on *ALS3* expression related to abiotic surfaces.

It is noteworthy that Nobile *et al*. [45] and Zhao *et al.* [47] have shown that *ALS3*-defective hyphal mutants of *C. albicans* are known to form disorganized, thin biofilms on catheter material. This was also the case for the biofilms formed by the HM strain in our study, as observed by SEM. Interestingly, however, we noted that the HM strain produced increased levels of *ALS3* transcripts in biofilms on human serum-treated SB discs than in serum-free controls at 90 min and 48 h.

Previous investigations have not revealed expression of *ALS3* transcripts in *C. albicans* biofilms “*in vivo”,* (in biofilms developed on catheters in live rats) although high expression of *ALS1* and *ALS2* has been observed [7]. It has been reported that certain *ALS* mutants of *C. albicans* cannot produce fully developed biofilms on silicone-elastomer catheter disks [11], the authors hypothesized that the different *ALS* genes may be active at different times and that they may complement each others’ functions.

### HWP1

The *HWP1* protein is expressed on the surface of germ tubes and hyphae but not during the yeast phase [51], and is required for filamentation, normal biofilm formation and virulence [45]. Furthermore, *HWP1* is a substrate for mammalian transglutaminase, which seems to form cross-links between *HWP1* and host-cell surface proteins *in vitro*, thus mediating stable attachment of hyphae to host epithelial cells [15].

We noted that *HWP1* transcript levels were much higher in WT planktonic cells than in HM planktonic cells. This result agrees with those of Sindl & Sundstrom, [52], who noted that *HWP1* mRNA levels might increase in the yeast form. Indeed, in the SEM analysis, *C. albicans,* which displays morphologically heterogenic forms (yeast, hyphae and pseudohyphae) [53], was abundant in planktonic phase cultures. This result corroborates the increased expression of the filament-inducing *HWP1* in these heterogeneic WT planktonic *Candida* cultures. *HWP1*, together with the hyphal proteins *ALS3* and *ALS1*, is an adhesin that might promote cell-cell or cell-substrate binding [54].

We also noted a dramatic decrease in *HWP1* transcript levels in both serum treated and untreated WT biofilms by 24 h. In the biofilm phase of the WT strain, hyphal-producing genes *HWP1* and *ECE1* were significantly downregulated during the experimental period, whereas these genes were not highly expressed in the HM strain.

### EAP1

In contrast to *ALS3* and *HWP1*, which are specifically expressed during hyphal development [54], *EAP1* is expressed in both yeast and hyphal cells [3], [16]. *EAP1* encodes a glycosyl-phosphatidylinositol-anchored, glucan-cross-linked cell wall protein that has a role in cell adhesion to surfaces. We observed much lower levels of *EAP1* expression at all three time points in HM planktonic cells than in WT planktonic cells. The decreased *EAP1* expression in HM planktonic cells might contribute to their apparently diminished cell-cell adhesion properties suggested by our SEM analysis. Nevertheless, *EAP1* expression in the biofilms of both strains was lower than in the corresponding planktonic cells in all the conditions tested, implying that *EAP1* may not be important for biofilm development, at least in this system.

### ECE1

*ECE1* expression has been shown to correlate with the extent of cell elongation [55]. In our study, *ECE1* mRNA levels were low during planktonic growth, although with moderate increases at 24 h and 48 h. WT strain on SB discs in the presence of human serum demonstrated an upregulation of *ECE1* during the adhesion phase and throughout the biofilm growth for 48 h, whereas the HM strain showed much lower expression levels of *ECE1* during the first 24 h to 48 h. Interestingly, the intensity of *ECE1* expression was observed to coincide with cell elongation morphologies seen on scanning electron micrographs of the WT and HM biofilms on serum coated SB discs.

Overall, expression of the adhesion genes in HM planktonic cells was generally lower than in WT planktonic cells. The reason for this low expression might be the fact that the HM strain lacks *EFG1*, a key transcriptional factor of the cAMP pathway that is essential for formation of hyphae [56]. *EFG1* is involved in the expression of *HWP1* and *SAP4 - SAP6* [22], [50], [57].

### SAP Gene Expression

In CVC-associated infection, site-specific co-regulated expression of enzymes, such as secreted aspartyl proteases (SAPs) and phospholipases (PLs), has a key role in yeast colonization [58], [59]. The various *SAP* genes (*SAP1*–*SAP10*) are differentially and selectively regulated in yeast and filamentous forms at different stages of infection, contributing to the virulence of the organism [60]. For example, *SAP1*–*SAP3* are predominantly expressed in yeast cells, whereas *SAP4*–*SAP6* are expressed in hyphae, the predominant cell type in *Candida* biofilms [61]. *SAP1* expression is also thought to contribute to the adherence of *C. albicans* [62], [63].

In the early planktonic phase (90 min), the WT strain exhibited significantly higher levels of all 10 *SAP* genes compared with the HM strain. However, this trend was reversed at 24 h, with HM showing higher expression levels for all 10 *SAP* genes compared with the WT strain. One possible explanation is that the upregulation of *SAP* genes in the HM strain could be partially compensating for the lack of the *EFG1* and *CPH1* genes, which encode two transcription factors required for hyphae formation. In particular, the upregulation of *SAP* genes could be contributing to adherence of the HM mutant, because it is known that the expression of *SAP1–SAP3* may contribute to *C. albicans* adherence [62], [63], *SAP4–SAP6* are expressed in the hyphal form [64], [65], which is known to be more adherent than the yeast form [21], [22]. It is noteworthy that *C. albicans* planktonic cultures consisted mostly of blastospores and slightly elongated yeast cells throughout the experimental period. Although most studies support the notion that the hyphal form contributes to virulence *per se*, several authors have noted that co-regulation of genes controlling hyphal morphogenesis with genes encoding other virulence factors confounds analysis [21], [66]. For instance, *SAP5* and *SAP6* have been described as being associated with the yeast-to-hypha morphological conversion, which is responsible for invasive infections [57]. Therefore, the formation of hyphae together with *SAP* expression may be a component of the overall virulence strategy of *C. albicans*.

Interestingly, we found that WT biofilms on serum-treated SB discs displayed an increased biofilm growth and showed higher levels of *SAP1–SAP3* and *SAP7–SAP10* transcripts than biofilms grown on untreated SB discs. Previous *in vitro* studies have shown that *SAP1–SAP3* contribute to the damage of host cells and tissues [67]–[69]. Although the roles of *SAP7–SAP10* in *C. albicans* infections are not fully understood [18], [60], both *SAP9* and *SAP10* expression are associated with fungal adherence, cell wall integrity and cell separation during budding [70]. In addition, it has been reported that *SAP7* is expressed in an intravenous infection model of candidiasis, thereby indicating that *SAP7* expression may be important in catheter infections [71]. Therefore, our findings, together with previous research, support the hypothesis that *SAP1–SAP3* and *SAP7–SAP10* may contribute to biofilm formation on silicone biomaterial.

### PL Gene Expression

We also investigated the expression of mRNA transcripts of *PL* genes in planktonic and biofilm cells. In a recent study, the expression levels of *PLB1* and *PLB2* were observed to be model-dependent [5]. The authors found low levels of *PLB1* and *PLB2* expression in biofilms grown on silicone in microtiter plates and on RHE (reconstituted human epithelium) up to 12 h, whereas *PLB2* was highly expressed in biofilms grown on RHE on silicone disks in a continuous flow system (the CDC reactor) [5]. In our study, *PLB1* and *PLB2* were upregulated in the WT planktonic cells; significant upregulation of both genes was also observed at 24 h in WT biofilms formed on human serum-coated SB discs (only *PLB1* was considerably upregulated at 48 h). The HM strain, however, demonstrated significant upregulation of *PLB1* in planktonic cultures and in biofilms incubated in human serum at all three time points. These results are in contrast to a previous study that reported that planktonic yeast cells produced more phospholipases than biofilm cells [72]. In summary, previous research have produced variable results regarding *PL* gene expression and our investigations also demonstrated that the different members of the *PL* gene family were expressed at different times in biofilms.

### Conclusions

Taken together, our results indicate that *in vitro* biofilm formation in *C. albi*cans, in the presence of human serum, is accompanied by variable and progressive changes in the expression of several virulence-related genes relative to incubation without human serum. Significant upregulation of virulence-related genes included those associated with adherence, hyphal growth and secretory aspartyl proteinases and phospholipases; *ALS3*, *HWP1*, *EAP1*, *ECE1*, *SAP1*, *SAP4*, *SAP6*–*SAP10*, *PLB1*, *PLB2* and *PLC*. However, the expression patterns of these virulence gene families under laboratory growth conditions do not seem to be representative of *in vivo* gene expression, which is probably attributable to the complex and variable environmental conditions in the host. Therefore, the roles of individual virulence genes during catheter infection need to be established in animal models using the respective gene-deficient candidal strains. The observations of this study, however, will be useful as fundamental data for such future studies.
